# Psychometric assessment of HIV stigma in patients attending a tertiary facility: An initial validation of the Berger HIV stigma scale in a Ghanaian perspective

**DOI:** 10.1371/journal.pone.0282193

**Published:** 2023-04-27

**Authors:** Nicholas Ekow Thomford, Fiifi Ndom Dampson, George Adjei, Sebastian Eliason, Martins Ekor, George Boateng Kyei

**Affiliations:** 1 Department of Medical Biochemistry, School of Medical Sciences, College of Health and Allied Sciences, University of Cape Coast, Cape Coast, Ghana; 2 Pharmacogenomics and Genomic Medicine Group, School of Medical Sciences, College of Health and Allied Sciences, University of Cape Coast, Cape Coast, Ghana; 3 Division of Human Genetics, Department of Pathology, Faculty of Health Sciences, University of Cape Town, Cape Town, South Africa; 4 Department of Community Medicine, School of Medical Sciences, College of Health and Allied Sciences, University of Cape Coast, Cape Coast, Ghana; 5 Department of Pharmacology, School of Medical Sciences, College of Health and Allied Sciences, University of Cape Coast, Cape Coast, Ghana; 6 Department of Virology, Noguchi Memorial Institute for Medical Research, College of Health Sciences, University of Ghana, Legon, Accra, Ghana; The Hong Kong Polytechnic University, HONG KONG

## Abstract

**Background:**

HIV-related stigma and discrimination are major challenges to people living with HIV (PLWHIV) and are due to misconceptions. Due to socioeconomic variations, there is increased stigma experienced by PLWHIV in sub-Saharan Africa (SSA). Stigma affects adherence to antiretroviral medications by PLWHIV and defeats the goal of achieving viral suppression. This study evaluated the Bergers HIV stigma scale in PLWHIV in Ghana regarding construct validity and reliability and assessed which aspect of stigma is critical for immediate redress.

**Methods:**

The Berger *et al*. HIV stigma scale (39 items) and some selected questions from HIV stigma and discrimination measurement tool of the International Centre for Research on Women, Washington, DC were administered to a cohort of PLWHIV in Ghana (n = 160). Clinico- demographic data was collected from their folders and verbally. The psychometric assessment included exploratory factor analysis whiles scale reliability was evaluated as internal consistency by calculating Cronbach’s α.

**Results:**

The exploratory factor analysis suggested a four-factor solution which is like the original Berger HIV scale with sub-scales *personalised stigma*, *disclosure concerns*, *negative self- image*, and concerns *with public attitudes*. Items in the sub-scales *personalised stigma* (15- items), *disclosure concerns* (6), *negative self-image* (7) and *concerns with public attitudes* (6) were reduced compared to the original scale. Cronbach’s α for the overall HIV stigma scale (34-items) was 0.808 whiles the sub-scales α ranged from 0.77 to 0.89. Analysis suggested the prevalence of a fundamental one-dimensional factor solution which yielded a 34-item scale after removing items for low factor loadings. Disclosure concerns was the highest ranked subscale although our study also found that about 65% of PLWHIV among our study participants had disclosed their status.

**Conclusion:**

Our 34-item abridged Berger HIV stigma scale showed sufficient reliability with high Cronbach’s α and construct validity. Disclosure concerns ranked high among the sub-scales on the scale. Exploring specific interventions and strategies to address stigma concerns in our population will aid in the reduction of HIV-related stigma and associated consequences.

## Introduction

Since the discovery of the Human Immuno-deficiency Virus (HIV), Africa has borne the brunt of the pandemic. HIV/AIDS continues to be a leading cause of disease burden in Africa at large and most especially sub-Saharan Africa (SSA) [1, 2]. Notwithstanding the successful implementation of antiretroviral therapies (ART) and significant decline in HIV/AIDS related mortality over the last two decades, 65% of new infections occur in SSA [2]. In the West Africa sub-region, Ghana has a low-level of HIV epidemic with disproportionately high prevalence in several key populations such as female sex workers (FSW) and men who have sex with men (MSM). Ghana is striving to achieve the revised UNAIDS agenda 95-95-95; however, there are key factors that can potentially derail the achievement of the agenda. One such key factor involves stigma experienced by HIV patients which can be enacted, anticipated and or internalised [3].

Stigma and discrimination are major challenges facing people living with HIV (PLWHIV) globally due to their HIV status. However, in SSA, this is worse compared to other jurisdictions [4–6]. A previous study exploring stigma at different stages of HIV care reported that presence of gossiping and absence of supportive structures increased cautious disclosure [7]. HIV stigma has a tremendous effect on disclosure and adherence to ARTs and therefore will defeat the goal of reducing infection and achieving viral suppression to undetectable levels [8, 9]. Ghana missed the target of the UNAIDS agenda 90-90-90 and many health workers have come to understand and believe that unless programmes are implemented to begin to address HIV-related stigma as well as treatment and management, overcoming the disease will be unsurmountable [10–12]. HIV stigma is considered as a social phenomenon, based on the categorisation and stereotyping of PLWHIV, influencing loss of status and discrimination [13, 14]. HIV related stigma is socially shared knowledge about the devalued status of PLWHIV [15]. This manifests into stereotyping, discrediting, discounting, prejudice and discrimination directed specifically at PLWHIV [13, 16]. This phenomenon may not be peculiar to Ghana alone as internationally, there has been a resurgence of interest in HIV and AIDS-related stigma and discrimination. To assess and measure stigma that can be translated into clinical practice at ART clinics, valid and reliable instruments are needed to map HIV stigma to develop country and population specific interventions [17]. Several stigma assessments instruments such as the internalised stigma scale [18], measures of stigma and social impact of diseases [19], enacted, vicarious, felt normative and internalized HIV stigma scales [15], stigma mechanisms of the HIV stigma framework [3] and the HIV stigma scale [20] have made efforts to elicit stigma in HIV patients. Each of these scales are evaluating an individual mechanism related to stigma. However, the HIV stigma scale by Berger *et al* [20] distinguishes between three stigma scales in a single instrument and yields an inclusive HIV stigma score as well as to the different stigma dimension scores. This instrument has been used to measure stigma in several populations including men and women in Kenya [21], African Americans [22], MSM, Nigeria [23] and Sweden [24]. Despite the robustness of the Berger *et al* HIV stigma scale, it may be tedious to administer in clinical practice especially considering resources that will be at the disposal of the health facility. For the various populations in which the Berger et al HIV stigma scale has been implemented, the 40-item instrument has mostly been shortened to make it population specific and focused for practical application [25–27]. In Ghana, HIV patients encounter several forms of stigma however, there is no instrument for measurement of HIV related stigma available in our context. Data from the original Berger HIV stigma scale suggests that the questionnaire be shortened. This study therefore set out to evaluate psychometric properties of stigma in HIV patients using the HIV stigma scale and develop a shortened version of the scale for translational application.

## Materials and methods

### Study population, recruitment, and consent

This cross-sectional study was conducted among patients presenting for care at the HIV clinic at the Cape Coast Teaching Hospital (CCTH), Cape Coast, Ghana between February and June 2022. The Cape Coast Teaching Hospital is a tertiary health facility with specialised clinics including a retro clinic that sees to PLWHIV. The retro clinic sees approximately 2000 patients annually. The characteristics of the patients at the retro clinic were considered representative regarding gender, period of diagnosis and reporting for periodic reviews. The inclusion criteria was: (1) Men and women diagnosed with HIV (2) aged 18 years and above (3) Consent to being part of the study. (4) ability to answer the questions in English or any local dialect. The research team was made up of clinicians, research nurses, final year medical students, medical scientists, and statisticians. The list of HIV patients that attend the retro clinic at the tertiary facility was used to select the participants where on the list every 10^th^ person was selected and contacted. The research nurse who is a member of the research team contacted eligible persons through telephone for their appointment dates at the retro clinic. Eligible participants were approached by a member of the research team when they came to the clinic for scheduled appointment and provided both verbal and written consent to participate in the study. The instrument was translated into the local dialect for those that could not read or speak English. All consenting participants who satisfied the selection criteria, completed a structured questionnaire that included clinicodemographic data, Berger HIV stigma scale and some selected questions from the measuring HIV stigma and discrimination tool culled from International Centre for Research on Women, Washington, DC [28]. In addition, the PLWHIV were asked about when they discovered their status and disclosures.

### Sample size determination

The prevalence of stigmatization among PLWHIV in a hospital-based study in Ghana was 12.1% [29]. Based on the data of the retro clinic in CCTH, the average total population is 2,106. With 95% confidence level, 5% margin of error and 5% provision for contingency the required sample size for the study is 160. The Raosoft software was used to calculate the sample size.

### Ethical consideration

This study was considered within the strictest of ethical standards as it involved patients who are considered as vulnerable. Written and verbal Informed consent was obtained from each participant and the study was explained in both English and the local languages. Anonymity was observed using codes that were generated for each participant. Data collected through computer assisted personal interview app Epicollect5 is highly protected, and access is limited. The study was approved by the ethical review board of the Cape Coast Teaching Hospital (CCTH/ERC/2020/109 and CCTH/ERC/2021/089).

## Data collection instruments

### Clinicodemographic characteristics

During the questionnaire administration, we collected both self-reported sex, gender, religion, ART adherence and marital status. We collected additional clinical data such as how long since they were diagnosed, HIV-status disclosure and medication from patient folders.

### The HIV stigma scale

The stigma instruments used were the Berger HIV-stigma scale and some selected questions from the measuring HIV stigma and discrimination tool. The Berger HIV scale is well validated globally. The instrument comprises of a 40-item questions that are sub-scaled with a 4-point likert type response ranging from “*strongly disagree*” to “*strongly agree*” [20]. The scale measures four factors which are personalised stigma, disclosure concerns, negative self- image, and concerns with public attitudes towards PLWHIV. The average time that it took to administer the questionnaires was 25–30 minutes depending on the literacy status of the participant.

### Data collection instrument

We employed a computer assisted personalised interview (CAPI) method to collect our data. This is a paperless procedure where questionnaires were loaded into Epicollect 5 [30] which is a mobile and web application developed by Imperial College, London and funded by Wellcome Trust. We created customized forms of the questionnaire for data collection. A strong password was created to secure the data collected and data was only accessible to Principal Investigators. Data collection was monitored in real time as and when they were uploaded to the server.

### Data analysis

A series of descriptive statistical analysis were undertaken to obtained characteristics for all study variables. We then conducted several analyses to explore the properties of the HIV sigma mechanism, given that this is the first time this scale is being used in our population.

Adopting a modified approach by Jayaseelan *et al* [31], the baseline clinicodemographic were collected as categorical variables and described using frequencies and percentages. An exploratory factor analysis (EFA) on the 39-item scale was undertaken to explore the latent nature of the dataset obtained. Randomised eigenvalues for parallel analysis were generated using the *factor* code in Stata. Eigenvalues greater than 1.0 from the principal component solution and screeplot from a principal factor solution with squared multiple correlations as estimates of communality were utilised to provide an indication of the number of underlying factors. An alpha factoring with oblique oblinim factor rotation for inter-factor correlations was used as the extraction method and the number of factors extracted was determined through parallel analysis as originally espoused by Berger et al [20]. The pattern matrix acquired was analysed using loadings and cut-off (≥0.6) for the loadings was used for subsequent analysis. We eliminated 5 of the 39 item that had low loadings of <0.6. The distribution of scores within the various subscales and parameters was evaluated using means and standard deviations (SDs). Mean scores were transformed linearly into a 0–100 range with higher scores representing higher levels of stigma. Item means and SDs were used as rough equivalent of the subscales and to justify item score summation into subscale scores. Intercorrelations were undertaken using the *pwcorr*, *sig*.*star (0*.*05*) code in Stata between various parameters and the Berger sub-scales. Statistical analyses were conducted using Stata software (version 17.0) (StataCorp, College Station,TX). All statistical tests were two-sided with p<0.05 considered as statistically significant.

### Data quality

Data quality was evaluated by analysing items with missing values or had no response. Should an item be found to have >3% of participants not answering, further interrogation would have been called for to understand what led to that. In this study however, it was observed that all participants answered all the items administered. Floor and ceiling effects which looks at the percentage of participants that scored the minimum (floor) and maximum (ceiling) for each item on the scale and subscale were considered acceptable ≤ 10%.

## Reliability

Reliability of Berger HIV scale in our population was assessed using Cronbach α, for the full scale and subscales to investigate internal consistency considered as sufficiently reliable if α≥0.7 [32].

## Results

### Characteristics of participants

Table 1 depicts the clinicodemographic data of study participants. This study involved 160 PLWHIV who attended a specialized retro clinic of a tertiary health facility in Ghana. Participants who agreed were made up of 29% males and 71% females with ages 18–79 years and mean age, 49.18 (SD = 12.61 years). Approximately ninety-six percent (96.25%) percent of the patients were on DTG/TDF/3TC regimen with 97% self-alluding to adherence. Approximately 97% had been diagnosed with HIV for at least 1 year and out of this percentage, 67% had disclosed their status to either their children, spouse, parents, or friends. For 76% of these patients, they learned of their status as part of laboratory testing for other suspected ailments; however, 10% mentioned they were coerced into doing the test although questionnaire did not follow up on who coerced them. Out of the 160 participants, approximately 24% had experienced some internalised stigma over the past 12 months concerning their status.

**Table 1:** Clinicodemographic characteristics of participants.

Characteristic	n (%)
**Gender**	
Male	47 (29.38)
Female	113 (70.62)
**Age** (**years**), mean ± SD	49.18 ± 12.61
18–19	3 (1.88)
20–39	31 (19.38)
40–59	90 (56.25)
60–79	36 (22.50)
**Education**	
Primary	83 (51.88)
JHS	61 (38.12)
Secondary	8 (5.00)
Tertiary	8 (5.00)
**Religion**	
Christian	156 (97.50)
Muslim	4 (2.50)
**Occupation**	
Artisan	9 (6.48)
Businessman	2 (1.44)
Businesswoman	1 (0.72)
Carpenter	2 (1.44)
Caterer	2 (1.44)
Cleaner	2 (1.44)
Cosmetologist	1 (0.72)
Farmer	24 (17.27)
Fisherman	3 (2.16)
Fishmonger	4 (2.88)
Seamstress	10 (7.19)
Security guard	2 (1.44)
Student	4 (2.88)
Teacher	2 (1.44)
Trader	67 (48.20)
Traditional birth attendant	1 (0.72)
Unemployed	2 (1.44)
**Marital status**	
Married	61 (38.12)
Widowed	31 (19.38)
Co-habiting	7 (4.38)
Single	13 (8.12)
Divorced	48 (30.00)
**ART medication**	
DTG/TDF/3TC	154 (96.25)
EFV/TDF/3TC	6 (3.75)
**When diagnosed with HIV**	
In the past 1 year	5 (3.12)
1 to 5 years ago	75 (46.88)
6 to 10 years ago	47 (29.38)
11–15 years ago	20 (12.50)
16–20 years ago	13 (8.12)
**Disclosure to other people**	
Spouse only	39 (24.38)
Children only	30 (18.75)
Friends only	2 (1.25)
Parents only	14 (8.75)
Children and Spouse	13 (8.13)
Parents and children	1 (0.63)
Spouse and Parents	4 (2.50)
Friends, Spouse and Parents	1 (0.63)
Pastor/Religious leader	1 (0.63)
Non-disclosure	55 (34.38)
**Decision to get tested**	
Sick and got tested	122 (76.25)
Coercion	16 (10.00)
Pregnant and got tested	12 (7.50)
Don’t remember	10 (6.25)
**Internalised stigma**	
***Feeling ashamed***	
Yes	29 (13.2)
No	139 (86.88)
***Feeling Guilty***	
Yes	24 (15.00)
No	136 (85.00)
***Self-blame***	
Yes	25 (15.72)
No	134 (84.28)
***Blame others***	
Yes	20 (12.50)
No	140 (87.50)
***Low self-esteem***	
Yes	26 (16.25)
No	134 (83.75)
***Suicidal***	
Yes	26 (16.25)
No	134 (83.75)

### Exploratory factor analysis

All participants answered the questions related to the Berger HIV-stigma scale and answers from the participants used for the exploratory factor analysis (EFA). EFA was undertaken to explore the latent structure of the data set. The adequacy of the data for factor analysis was investigated using the Kaiser-Meyer-Olkin measure for sampling adequacy and the data was found to be suitable for EFA, with a Kaiser–Meyer–Olkin test of 0.901. There were no floor and ceiling effects. Parallel analysis and screeplot showed a four-factor solution with 34 items clustering with loadings ≥0.6. Each item of the remaining 34 items was assigned to a single factor according to their highest loadings. Construct validity for the various sub-scales was therefore proven. Five items had loadings of <0.6 and were excluded in further analysis. These were: Item 11 -*“It is easier to avoid new friendships than worry about telling someone that I have HIV”*, item 23 -*“Having HIV in my body is disgusting to me”*, item 19- *“Since learning I have HIV*, *I worry about people discriminating against me”*, item 25 - *“I worry that people who know I have HIV will tell others”* and item 30 -*“People have told me that getting HIV is what I deserve for how I lived my life”*. Factor loadings for items with loadings >0.6 which were included for further analysis are shown in **Table 2**.

**Table 2:** Factor loadings >0.6 for items in the HIV stigma scale.

Items	Factor 1	Factor 2	Factor 3	Factor 4
1. In many areas of my life, no one knows that I have HIV		0.8833		
2. I feel guilty because I have HIV				0.6095
3. People’s attitudes about HIV make me feel worse about myself				0.7534
5. People with HIV lose their jobs when their employers find out				0.7248
6. I work hard to keep my HIV a secret		0.8364		
7. I feel I am not as good a person as others because I have HIV	0.9677			
8. I never feel ashamed of having HI(R)				0.8811
9. People with HIV are treated like outcasts				0.8088
10. Most people believe that a person who has HIV is dirty				0.7773
12. Having HIV makes me feel unclean				
13. Since learning I have HIV; I feel set apart and isolated from the rest of the world				0.8811
14. Most people think that a person with HIV is disgusting	0.9441			
15. Having HIV makes me feel that I’m a bad person	0.9441			
16. Most people with HIV are rejected when others find out				0.6153
17. I am very careful who I tell that I have HIV		0.8397		
18. Some people who know that I have HIV have grown more distant	0.8748			
20. Most people are uncomfortable around someone with HIV			0.6486	
21. I never feel the need to hide the fact that I have HIV				0.9677
22. I worry that people may judge me when they learn I have HIV		0.8067		
24. I have been hurt by how people reacted to learning I have HIV	0.7717			
26. I regret having told some people that I have HIV			0.6107	
27. As a rule, telling others that I have HIV has been a mistake			0.7570	
28. Some people avoid touching me once they know I have HIV	0.6314			
29. People I care about stopped calling after learning I have HIV	0.9180			
31. Some people close to me are afraid others will reject them if it becomes known that I have HIV			0.7409	
32. People don’t want me around their children once they know I have HIV	0.6024			
33. People have physically backed away from me when they learn I have HIV	0.9448			
34. Some people act as though it’s my fault I have HIV			0.7291	
35. I have stopped socializing with some people because of their reactions to my having HIV	0.8347			
36. I have lost friends by telling them I have HIV				0.7349
37. I have told people close to me to keep the fact that I have HIV a Secret		0.8060		
38. People who know I have HIV tend to ignore my good points	0.8118			
39. People seem afraid of me once they learn I have HIV	0.9448			
40. When people learn you have HIV, they look for flaws in your Character				0.8613

A, Rotated factor loadings (pattern matrix) based on alpha factoring. Factor loadings <0.6 not included

(R) indicates reverse scored items as in the original Berger et al scale

### Shortened scale and data quality

The breakdown of the response from participants is shown in **Table 3**. For all items of the Berger HIV stigma scale, there were no missing values or unanswered questions. After the EFA, the items in the sub-scales had reduced in comparison to the original sub-scales by Berger [20]. Per the item response scoring, mean scores for responses to personalised stigma and negative self-image hovered between disagree and agree (scale 2–3), while mean scores for disclosure concerns and concerns with public attitudes were around agree (scale 3).

**Table 3:** Descriptive analysis of items with loading factor >0.6 in the HIV stigma scale.

Items	N	Strongly disagree	Disagree	Agree	Strongly agree	Mean score ± SD
**Personalised stigma**						
18. Some people who know that I have HIV have grown more distant	160	4 (2.50)	149 (93.12)	7 (4.38)	0 (0.00)	2.09 ± 0.26
24. I have been hurt by how people reacted to learning I have HIV	160	1 (0.62)	154 (96.25)	5 (3.12)	0 (0.000	2.03 ± 0.19
26. I regret having told some people that I have HIV	160	5 (3.12)	143 (89.38)	12 (7.50)	0 (0.00)	2.04 ± 0.32
27. As a rule, telling others that I have HIV has been a mistake	160	12 (7.50)	125 (78.12)	16 (10.00)	7 (4.38)	2.11 ± 0.58
28. Some people avoid touching me once they know I have HIV	160		154 (96.25)	6 (3.75)		2.03 ± 0.19
29. People I care about stopped calling after learning I have HIV	160	6 (3.75)	148 (92.50)	6 (3.75)	0 (0.00)	2.00 ± 0.27
31. Some people close to me are afraid others will reject them if it becomes known that I have HIV	160	7 (4.38)	132 (82.50)	20 (12.50)	1 (0.62)	2.09 ± 0.43
32. People don’t want me around their children once they know I have HIV	160	7 (4.38)	145 (90.62)	7 (4.38)	1 (0.62)	2.01 ± 0.34
33. People have physically backed away from me when they learn I have HIV	160	6 (3.77)	147 (92.45)	6 (3.77)	0 (0.00)	2.00 ± 0.28
34. Some people act as ‘though it’s my fault I have HIV	160	12 (7.50)	133 (83.12)	15 (9.38)	0 (0.00)	2.02 ± 0.41
35. I have stopped socializing with some people because of their reactions to my having HIV	160	3 (1.88)	153 (95.62)	4 (2.50)	0 (0.00)	2.01 ± 0.20
36. I have lost friends by telling them I have HIV	160	1 (0.62)	154 (96.25)	3 (1.88)	2 (1.25)	2.04 ± 0.27
38. People who know I have HIV tend to ignore my good points	160	5 (3.12)	147 (91.88)	8 (5.00)	0 (0.00)	2.09 ± 0.29
39. People seem afraid of me once they learn I have HIV	160	6 (3.75)	148 (92.50)	6 (3.75)	0 (0.00)	2.00 ± 0.27
40. When people learn you have HIV, they look for flaws in your character	160	1 (0.62)	9 (5.62)	140 (87.50)	10 (6.25)	2.99 ±0.38
**Disclosure concerns**						
1. In many areas of my life, no one knows that I have HIV	160	7 (4.38)	1 (0.62)	55 (34.38)	97 (60.62)	3.51 ± 0.72
6. I work hard to keep my HIV a secret	160	7 (4.38)	2 (1.25)	71 (44.38)	80 (50.00)	3.4 ± 0.773
17. I am very careful who I tell that I have HIV	160	7 (4.38)	2 (1.25)	75 (46.88)	76 (47.50)	3.38 ± 0.72
21. I never feel the need to hide the fact that I have HIV (R)	160	15 (9.38)	143 (89.38)	2 (1.25)	0 (0.00)	3.08 ± 0.31
22. I worry that people may judge me when they learn I have HIV	160	6 (3.75)	1 (0.62)	94 (58.75)	59 (36.88)	3.28 ± 0.67
37. I have told people close to me to keep the fact that I have HIV a secret	160	6 (3.75)	9 (5.62)	93 (58.12)	52 (32.50)	3.19 ± 0.70
**Negative self-image**						
2. I feel guilty because I have HIV	160	0 (0.00)	136 (85.00)	24 (15.00)	0 (0.00)	2.15 ± 0.3’
3. People’s attitudes about HIV make me feel worse about myself	160	6 (3.75)	121 (75.62)	29 (18.12)	4 (2.50)	2.19 ± 0.53
7. I feel I am not as good a person as others because I have HIV	160	7 (4.38)	103 (64.38)	44 (27.50)	6 (3.75)	2.31 ± 0.61
8. I never feel ashamed of Iiving HIV (R)	160	15 (9.38)	143 (89.38)	2 (1.25)	0 (0.00)	3.08 ± 0.32
12. Having HIV makes me feel unclean	160	5 (3.12)	99 (61.88)	46 (28.75)	10 (6.25)	2.38 ± 0.65
13. Since learning I have HIV, I feel set apart and isolated from the rest of the world	160	8 (5.00)	127 (579.38)	24 (15.00)	1 (0.62)	2.11 ± 0.46
15. Having HIV makes me feel that I’m a bad person	160	5 (3.12)	99 (61.88)	46 (28.75)	10 (6.25)	2.38 ± 0.65
**Concerns with public attitudes**						
5. People with HIV lose their jobs when their employers find out	160	0 (0.00)	15 (9.38)	129 (80.62)	16 (10.00)	3.00 ± 0.44
9. People with HIV are treated like outcasts	160	0 (0.00)	7 (4.38)	142 (88.75)	11 (6.88)	3.03 ± 0.34
10. Most people believe that a person who has HIV is dirty	160	3 (1.88)	12 (7.50)	128 (80.00)	17 (10.62)	2.99 ± 0.51
14. Most people think that a person with HIV is disgusting	160	0 (0.00)	8 (5.000	133 (83.12)	19 (11.88)	3.06 ± 0.41
16. Most people with HIV are rejected when others find out	160	1 (0.62)	7 (4.38)	131 (81.88)	21 (13.12)	3.08 ± 0.44
20. Most people are uncomfortable around someone with HIV	160	2 (1.25)	6 (3.75)	142 (88.75)	10 (6.25)	3.00 ± 0.39

Descriptive statistics for the overall HIV stigma scale and subscales are shown in **Table 4**. Among the subscales disclosure concerns contributed the most with highest possible score of 82.70%, while personalised stigma contributed the least with a score of 52.37%. Cronbach’s α for the subscales ranged from 0.771 to 0.892 with the total for the overall scale (34 items) generating an α of 0.808.

**Table 4:** Reliability coefficients for overall HIV stigma scale and sub-scales.

Sub-scale (n of items)	Possible range	Means	% Highest possible score^a^	Reliability α
**HIV stigma–scale (34)**	**34–136**	**88.97 ± 6.51**	**66.42**	**0.808**
*Personalised stigma (15)*	15–60	31.42 ± 3.11	52.37	0.892
*Disclosure concerns (6)*	6–24	19.85 ± 3.08	82.70	0.869
*Negative self-image (7)*	7–28	16.61± 2.35	59.32	0.771
*Concerns with public attitudes (6)*	6–24	18.17 ± 1.82	75.71	0.784

a % highest possible score = (mean score/ upper limit of expected score range) × 100. The higher the % score, the higher the level of stigma and vice versa

Table 5 shows the correlations and intercorrelation between clinicodemographic, internalized stigma and Berger sub-scale. There was low correlations between clinicodemographic and the Berger subscales. Gender and disclosure concerns were negatively correlated (r = —0.17), whiles age was negatively correlated with personalized stigma and negative self-image (r = -0.16 and r = -0.28 respectively). There was significant positive correlation between medication and personalized stigma (r = -0.16). Internalised stigma also correlated well with the various subscales with negative correlations observed between feeling ashamed and disclosure concerns and negative self-image (r = -0.20 and r = 0.33), feeling guilty and disclosure concerns (r = -0.24), and low self-esteem and disclosure concerns (r = -0.22). Significant positive correlations were observed between personalized stigma and feeling guilty, self-blame, low self-esteem and feeling suicidal (r-0.28, r = 0.34, r = 0.32 and r = 0.30 respectively), negative self-image and feeling ashamed, feeling guilty, self-blame, low self-esteem and feeling suicidal (r = 0.33, r = 0.45, r = 0.37, r = 0.44 and r = 0.38 respectively) whiles blaming others and concerns with public attitudes significantly correlated (r = 0.27). As expected, intercorrelations between the sub-scales showed positive correlations between negatively self-image and personalized stigma (r = 0.390), concerns with public attitude and disclosure concerns and negative self-image (r = 0.34 and r-0.26 respectively). A low negative correlation was observed between disclosure concerns and personalized stigma (r = -0.02).

**Table 5:** Correlations between demographics, internalised stigma and Berger scale (n = 160).

	Personalised stigma	Disclosure concerns	Negative self-image	Concerns with public attitudes
**Clinicodemographic**				
*Gender*	0.01	-0.17*	0.07	-0.10
*Religion*	-0.02	-0.02	0.01	0.07
*Education*	0.11	-0.03	0.10	0.03
*Occupation*	0.08	-0.01	-0.04	-0.04
*Marital Status*	0.15	0.02	0.18*	0.09
*Age*	-0.16*	0.06	-0.28*	-0.13
*Medication*	0.16*	-0.13	-0.01	0.04
**Internalised stigma**				
*Feeling ashamed*	0.15	-0.20*	0.33*	0.04
*Feeling Guilty*	0.28*	-0.24*	0.45*	0.02
*Self-blame*	0.34*	-0.13	0.37*	0.03
*Blame others*	0.06	0.12	0.10	0.27*
*Low self-esteem*	0.32*	-0.22*	0.44*	0.04
*Suicidal*	0.30*	-0.07	0.38*	0.13
**Berger scale**				
*Personalised stigma*				
*Disclosure concerns*	-0.02			
*Negative self-image*	0.39*	0.05		
*Concerns with public attitudes*	0.01	0.34*	0.26*	

## Discussion

HIV-related stigma is well documented though measuring stigma is mostly arbitrary. PLWHIV who experience stigma live their lives with efforts to keep their status to themselves or to a few people as possible. HIV-related stigma limits prevention which also leads to poor medication adherence, non-disclosure, depression, suicidal thoughts rejection and isolation [33–35]. The clinicodemographic data obtained from this study shows that, out of a combined total of 46% of our participants who were married or co-habiting, only 24% had informed their live-in partner (spouse) of their HIV status (Table 1). In this study, 76% of the participants indicated they learned of their HIV status only when they had fallen sick and as part of laboratory tests while a significant 10% mentioned they were coerced into taking HIV test to discover their status. Coercion may take several forms including physical means which could manifest in the form of actual violence or threatening to take away a valuable thing if the person did not take a test such as losing a job or not having sex [36]. The World Health Organisation (WHO) in its 2016 guidelines on HIV self-testing explicitly frowns on using coercive means for people to test. Coercion can significantly influence HIV-related stigma. Approximately 15% of the participants had experienced some form of internalised stigma over the past 12 months before questionnaire administration. Internalised stigma had been reported in several PLWHIV in places such as Bangladesh [37], New York [38], Cambodia, the Dominican Republic, Uganda, Tanzania [39] and South Africa [40]. Internalised stigma contributes significantly to depression, anxiety, and hopelessness even in the face effective ARTs that improves quality of life for PLWHIV.

In trying to standardise tools of measurement for HIV-related stigma, this study sought to evaluate the Berger HIV stigma scale in a context involving HIV positive men and women as a psychometric measure. Using psychometric techniques, we evaluated the well-known Berger HIV stigma scale in a cohort of Ghanaians. After excluding about 5 factors in all, due to low loadings after starting with 39 items from the original Berger HIV stigma scale, the instrument was shown to have adequate construct validity and reliability. The original Berger HIV scale is a 40-item instrument that is useful and relevant to our context. The instrument, however, can be tedious to administer, and potentially translate it into clinical practice so as part of recommendations from the other users or implementers of the scale, there should be validation and potential abridgement undertaken to see to apt utilisation of the scale. The HIV stigma scale was reduced to a 34-item instrument that were sub-scaled into the original personalised stigma with 15 items, disclosure concerns with 6 items, negative self-image with 7 items and concerns with public attitudes with 6 items. The number of items which make up each sub-scale is different from what has been published in the Swedish context [24], South India [31], Vermont and Northern England [41] and the original Berger HIV stigma scale [20]. Reliability and internal consistency (Cronbach’s α) for the overall 34-item scale and sub-scales was greater than the 0.70 benchmark [42, 43] ranging from 0.771 to 0.892. The Cronbach’s α obtained is consistent with other similar studies that sought to reduce the Berger scale to shorter versions including the original scale. The scores obtained for the 34-item instrument shows an overall perceived HIV-stigma scale mean score of 88.97 ± 6.51 and this is at variance with other studies that have described high rate of HIV-related stigma among PLWHIV [44, 45]. These studies reported higher mean overall stigma scores of over 95 which is also influenced by the number of items in the instrument used. Based on the possible scale range and considering the minimum and maximum scores, we can say that there is generally a moderate perceived stigma among the participants although there could be more intense experienced stigma. For one to disclose his or her status to someone, then it is expected that the person to whom the PLWHIV discloses to will keep whatever that is heard on the health status of the person confidential. PLWHIV are likely to disclose their status to someone they can trust and in an environment that is less judgemental or low stigma with no or less negative consequences [46]. Several studies have reported non-disclosure of HIV status among PLWHIV due to severe stigma [47, 48]. Analysis of the 4 subscales involving different items showed disclosure concerns had the highest possible score of 82.7%. This is consistent with other studies that have used the Berger HIV scale [22, 24, 44]. Disclosure of status has significant effects on achieving the agenda UNAIDS 95-95-95 as such PLWHIV can be offered the needed assistance in counselling, medication, and monitoring. Although 67% had disclosed their status to their children, spouse, parents, or friends, the findings from the study suggest disclosure is still seen as a concern. The plausible explanation for these findings could be concerns of those disclosed who may not be able to keep it a secret or may disclose it at some point in time consciously or unconsciously. Disclosure is considered a way to open up and a critical step in bringing an end to stigma and discrimination against PLWHIV [49]. Often times, there are challenges with involuntary disclosure by relatives, partners or even health workers. For health workers, the issue of involuntary disclosure has to do in part to lack of private spaces and difficulty in balancing medical confidentiality [50].

Negative self-image and concerns with public attitude had prevalent scores of 59.32% and 75.71% respectively. Negative self-image has been found to be associated with depression, drug abuse and alcohol misuse which is unhealthy for PLWHIV [51]. Alcohol misuse and drug abuse further exacerbate commitment to HIV care (PLWHIV), health outcomes involving ART adherence and subsequent viral suppression and mental health. Future studies should look at negative self-image and alcohol and drug abuse as a coping mechanism in PLWHIV. Issues of concerns with public attitudes may be one reason why non-disclosure is high especially in resource limited settings. Concerns with public attitudes had the second highest subscale score which is like other studies previously mentioned. Public education on HIV should be intensified to help reduce stigmatisation. Community organisations should be formed to share lived experiences, collective identities, and education on HIV which creates connections and inspires community awareness ameliorating stigma. The ripple effect is that once HIV infected persons have a “comfortable” public space, disclosure will increase which will be beneficial in achieving the UNAIDS agenda 95-95-95. Our unpublished data from one of our studies shows significant efforts must be made in order to get PLWHIV to come for review and even take their ART refills. Dedicated spaces to health facilities are practically known to all as HIV clinics so anyone who enters and leaves that place is classified as a patient which makes PLWHIV uncomfortable and therefore skip their appointments.

Personalized stigma was the lowest prevalent subscale of the HIV stigma scale in our study which is in line with other studies conducted [23, 52]. However, other studies have also found it to be a prevalent issue including one study that revealed that personalised stigma is a prevalent issue in Florida, USA especially among women and non-White Latinos [53] and one that found out that perceived public and experienced stigma were mediated by self-stigma [54]. Personalised stigma has significant effects on self-esteem which affects quality of life of PLWHIV.

There was low correlation between clinicodemographic, internalized stigma scale and Berger scale assessing the various parameters. Though the magnitude of correlations were generally ranging r = -0.01 to r = 45, there were significant correlations among the various parameters analysed. This is similar to a previous study conducted in stigma subscales where there were low correlations (r = 17 to 0.46) [55].

## Limitations

There are some limitations worth highlighting in this study. First, due to the cross-sectional nature of this study, causal relationships between individual characteristics and HIV-related stigma could not be determined. Secondly, all indicators were self-reported which may underestimate the accurate experience of the stigma due to social appropriateness bias. The intensity of emotions and/or feelings at the time of the study, PLWHIV experienced stigma might have dwindled. The HIV stigma scale may have temporality concerns as changes in stigma over a time may be missed.

## Conclusion

HIV-related stigma can be found in the Ghanaian society. Our findings show moderate levels of stigma in PLWHIV which might be attributed to the high level of disclosure by participants. Despite the high level of disclosure, disclosure concerns ranked as the highest HIV stigma subscale with personalised stigma being the least ranked. Coercion and routine laboratory were ways in which our participants discovered their HIV status, and these can lead to some form of stigma. Exploring specific interventions and strategies such as community education and individual needs may help reduce HIV-related stigma.

## Supporting information

S1 File (CSV)
